# Prevalence and risk factors of malnutrition in elderly patients with diabetes mellitus: a meta-analysis and systematic review

**DOI:** 10.3389/fnut.2026.1783774

**Published:** 2026-08-10

**Authors:** Lingling Tang, Yushan Shen, Run Li, Jiajia Jia, Fangmin Cao

**Affiliations:** 1 Department of Endocrinology and Metabolism, The Second People’s Hospital of Yibin, Yibin, Sichuan, China; 2 The Medical Specialist Alliance for Endocrinology and Metabolism of Yibin, Yibin, Sichuan, China; 3 The Affiliated Traditional Chinese Medicine Hospital, Southwest Medical University, Luzhou, Sichuan, China; 4 Compounded Infusion Quality Testing Analysis and Precision Medication Technological Innovation Key Laboratory of Luzhou, Luzhou, Sichuan, China; 5 School of Nursing, Southwest Medical University, Luzhou, Sichuan, China

**Keywords:** diabetes, elderly, malnutrition, meta-analysis, prevalence, risk factors

## Abstract

**Background:**

The prevalence and risk factors of malnutrition in elderly patients with diabetes are unclear, a knowledge gap that this meta-analysis aimed to explore.

**Methods:**

Studies published in the PubMed, Embase, Web of Science, Medline, EBSCO, CNKI, Wanfang, VIP, and Sinomed databases from 1 January 2010, to 31 December 2024 were searched using the following terms with a combination of subject headings and free text: “diabetes,” “malnutrition,” “prevalence,” “risk factors,” “associated factor,” and “influencing factor.” Age filters (age ≥ 60 years) were applied where available. All retrieved studies were manually screened for age criteria. The term “elderly” was not used as a search term to avoid missing studies that enrolled older adults without mentioning “elderly” in the title or abstract. Cohort studies, case–control studies, and cross-sectional studies meeting the inclusion criteria were analyzed. Meta-analysis was performed using Stata 15.0 software.

**Results:**

This meta-analysis included 30 studies involving 12,564 cases from 9 countries (namely China, South Korea, Japan, Thailand, Turkey, Nigeria, Spain, Brazil, and India). Based on 21 studies, the overall prevalence rate of malnutrition in elderly patients with diabetes was 29% (95% confidence interval (CI): 0.19–0.39). The analysis of 20 studies revealed that the prevalence rate of high malnutrition risk was 42% (95% CI: 0.31–0.53). This study identified 14 risk factors for malnutrition in elderly patients with diabetes.

**Conclusion:**

This meta-analysis improved our understanding of the prevalence and risk factors of malnutrition in elderly patients with diabetes.

**Systematic review registration:**

https://www.crd.york.ac.uk/PROSPERO/view/CRD420251034655, identifier CRD420251034655.

## 1 Introduction

As of 2024, the global prevalence of diabetes among adults is estimated to exceed 830 million. China reported a diabetes prevalence rate of 12.3–13.1% depending on diagnostic criteria (1, 2). Among individuals aged ≥ 60 years, the prevalence of diabetes is > 20%. By 2050, diabetes is projected to affect more than 1.3 billion individuals worldwide (3, 4). In addition to the risk of diabetic complications, patients with diabetes are prone to malnutrition due to declining physiological function and lack of social support (5). In particular, elderly patients with diabetes are susceptible to disease-related weight loss, reduced muscle mass and strength, and frailty syndrome, adversely affecting disease recovery and clinical outcomes (6, 7). Malnutrition is characterized by insufficient, excessive, or unbalanced nutritional intake, leading to weight loss, nutrient deficiencies, and other symptoms affecting the overall health (8, 9).

The impact of malnutrition in the elderly is more severe than that in the younger population. Compared to younger individuals, older adults experience increased changes in body composition during malnutrition (10). Elderly patients with diabetes and malnutrition exhibit poor prognosis, including enhanced disability and mortality rates and prolonged hospital stays (11, 12). Malnutrition can also lead to insulin resistance and *β*-cell dysfunction, leading to the development of complex metabolic disorders (13, 14). Additionally, malnutrition impairs the recovery of body function and increases the risk of mortality and readmission (15, 16). Strict dietary restriction and polypharmacy, which are common diabetes management practices, may directly induce or exacerbate malnutrition. Excessive restriction of carbohydrates and protein can lead to energy and essential nutrient deficiencies. Polypharmacy—the concomitant usage of ≥ 5 medications—is common among elderly patients owing to multiple comorbidities. Drugs, such as metformin (which causes gastrointestinal disturbance and vitamin B₁₂ malabsorption), cardiovascular agents (such as digoxin and amiodarone, which cause anorexia and taste alteration), and anticholinergic drugs (which cause dry mouth and constipation), can impair appetite, nutrient absorption, and physical function. Therefore, the prevalence and related risk factors of malnutrition in elderly patients with diabetes must be clarified to enable timely nutritional interventions and improve prognosis.

Previous studies have reported differential prevalence rates and risk factors of malnutrition in elderly patients with diabetes (17). This can be attributed to different nutritional assessment tools (such as Mini Nutritional Assessment [MNA], MNA-Short Form [MNA-SF], Nutritional Risk Screening 2002 [NRS-2002], and Geriatric Nutritional Risk Index [GNRI]) with varying sensitivity and specificity, study settings (hospital, community, and nursing home), geographic locations (Asia, Europe, Africa, and South America), and clinical features, such as chronic complications, polypharmacy, and diabetes duration (17). The underestimation of malnutrition prevalence can prevent the identification of malnutrition by healthcare providers, contributing to poor prognosis. This study performed a systematic review and meta-analysis to summarize the prevalence of malnutrition and high malnutrition risk in elderly patients with diabetes, analyze related risk factors, and provide a reference for early nutritional intervention in elderly patients with diabetes to improve the quality of life and mitigate family and societal burden.

## 2 Data and methods

### 2.1 Agreement and registration

This meta-analysis was performed following the Preferred Reporting Items for Systematic Reviews and Meta-Analyses guidelines. The study protocol was registered in the International Prospective Register of Systematic Reviews (PROSPERO) (ID: CRD420251034655).

### 2.2 Inclusion and exclusion criteria

The inclusion criteria were based on the Population, Intervention, Comparison, and Outcome framework.

#### 2.2.1 Inclusion criteria

The inclusion criteria were as follows: cohort, case–control, or cross-sectional studies; studies involving patients with diabetes aged ≥ 60 years with or without chronic diabetic complications, irrespective of ethnicity or nationality; studies examining malnutrition or high malnutrition risk; studies analyzing the prevalence of malnutrition or high malnutrition risk and the influencing factors of malnutrition.

#### 2.2.2 Exclusion criteria

The exclusion criteria were as follows: studies that diagnosed malnutrition based solely on low body mass index (BMI); studies with a sample size of < 50; duplicate publications (only the first published version was retained); studies with overlapping data; studies involving patients undergoing peritoneal dialysis or those with cancer, tuberculosis, or other diseases severely affecting nutritional status; review articles and conference abstracts.

### 2.3 Search strategy

A combination of subject headings and free words with Boolean operators (OR/AND) was used to search for literature published between 1 January 2010, and 31 December 2024, in nine databases (PubMed, Embase, Web of Science, Medline, EBSCO, CNKI, Wanfang, VIP, and Sinomed). The search strategy combined MeSH/Emtree terms and free-text words for “diabetes mellitus,” “malnutrition,” “prevalence,” “risk factor,” “associated factor,” and “influencing factor.” To focus on the elderly population, age filters (age ≥ 60 years) were applied where applicable. All retrieved studies were manually screened for age criteria. The term “elderly” was not used as a search term to avoid missing studies that enrolled older adults without mentioning the term “elderly” in titles or abstracts. The reference lists of included studies were also tracked. Detailed search strategies are provided in Supplementary Table S1.

### 2.4 Literature screening and data extraction

Two investigators independently screened the literature and cross-checked the results. Disagreements were resolved via discussion with a third investigator. The following data were extracted using pre-designed tables: first author name, publication year, study type, study site, country, sample size, gender, age, and assessment tool, prevalence of malnutrition or high malnutrition risk, and influencing factors.

### 2.5 Quality assessment

Two evaluators independently assessed the quality. Disagreements were resolved based on third-party inputs. Cross-sectional studies were evaluated using the Agency for Healthcare Research and Quality (AHRQ) criteria (11 items; yes = 1 point, no/unclear = 0; item 5 reverse-scored; maximum 11 points; ≥ 8 points = high quality, 6–7 points = medium quality, ≤ 5 points = low quality). Cohort and case–control studies were assessed using the Newcastle-Ottawa Scale (NOS) (maximum 9 points; 0–3 points = low quality, 4–6 points = medium quality, 7–9 points = high quality). Low-quality studies were excluded.

### 2.6 Statistical analysis

Meta-analysis was performed using Stata 15.0. Categorical and continuous data were analyzed using odds ratios (OR) and mean differences, respectively, along with 95% confidence interval (CI) values. Heterogeneity was assessed using *I*^2^ and the chi-squared test. The criteria for significant heterogeneity were as follows: *I*^2^ ≥ 50% and *p* < 0.1. A random-effects model was used. Subgroup analyses were performed for categorical variables, with comparisons performed using chi-squared tests. Sensitivity analysis was performed by sequentially excluding each study. Publication bias was evaluated through visual inspection of funnel plots and Egger’s test (18). Differences were considered statistically significant at *p* < 0.05.

## 3 Results

### 3.1 Study selection

**Figure 1:**
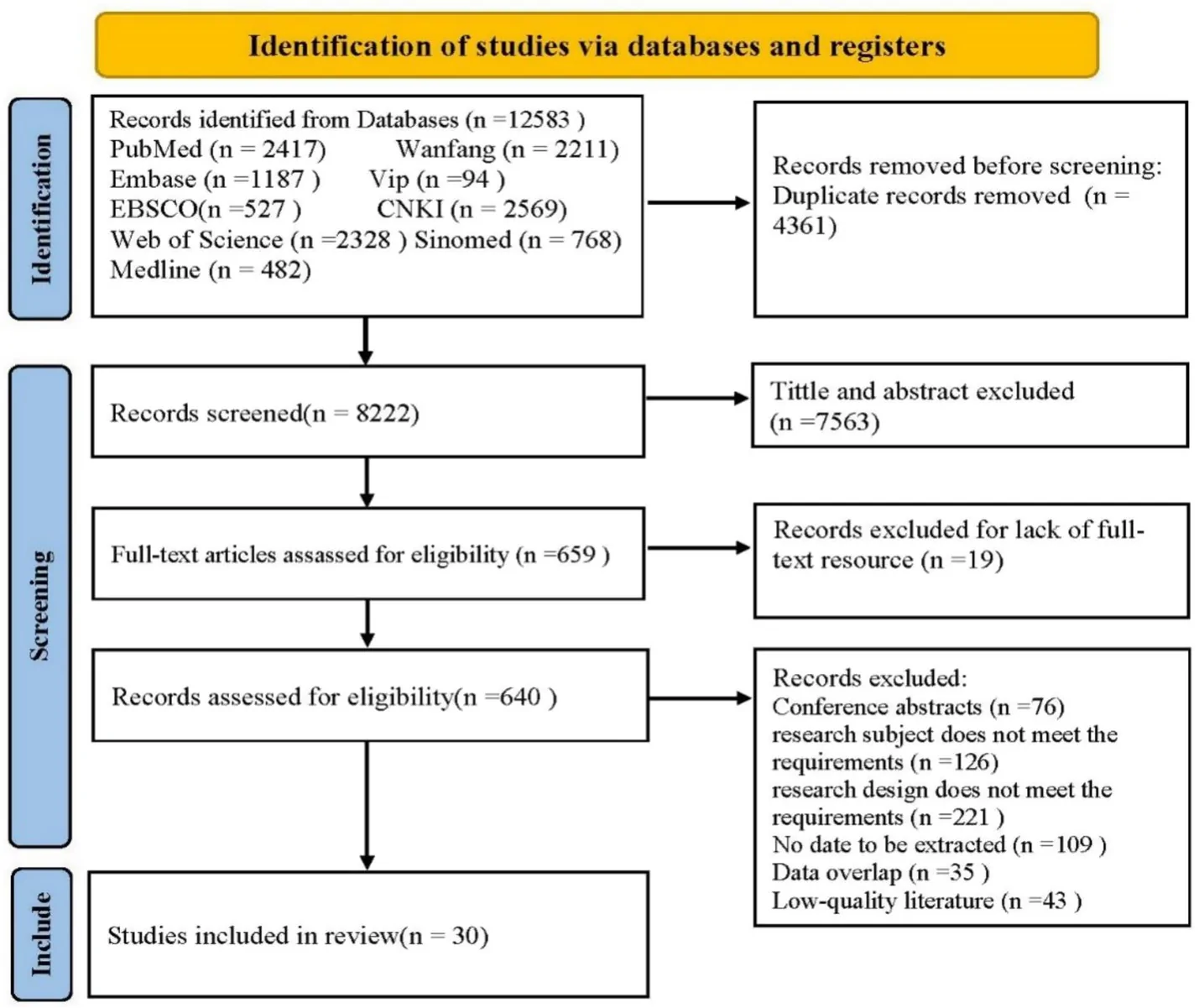
Flow chart for selecting the studies.

This study retrieved 12,583 articles. Among these, 4,361 duplicates were removed. After screening titles, abstracts, and full texts, 30 studies from 9 countries (namely China, South Korea, Japan, Thailand, Turkey, Nigeria, Spain, Brazil, and India) involving 12,564 cases were finally included. The literature screening process is shown in Figure 1.

### 3.2 Characteristics of included studies

The design of the included studies was as follows: cross-sectional studies (*n* = 26) (19–44); cohort studies (*n* = 2) (45, 46); case–control studies (*n* = 2) (47, 48). The included studies used the following seven nutritional assessment tools: MNA; MNA-Short Form (MNA-SF); Nutritional Risk Screening 2002 (NRS-2002); GNRI; Global Leadership Initiative on Malnutrition; Nutrition Screening Initiative (NSI); DETERMINE Your Nutritional Health Checklist. The main characteristics of the included studies are shown in Table 1.

**Table 1:** Characteristic of included studies.

Author (year)	Study country	Study design	Sample (n)	Participants	Age, (year)	Measurement tools	Research location	Proportion of women (%)	Prevalence (%)		Risk factors
									Malnutrition^a^	Risk^b^	
Gao et al. (19)	China	Cross-sectional	252	T2DM	≥65	MNA-SF	Inpatient Department	50.0	7.1	49.6	NA
Ling et al. (20)	China	Cross-sectional	382	T2DM	≥60	MNA-SF	Outpatient Department	40.8	22.25	—	① ② ③ ④ ⑤ ⑥ ⑭
Chu et al. (21)	China	Cross-sectional	426	T2DM	≥65	MNA-SF	Inpatient Department	36.3	19.25	36.85	① ② ④ ⑤ ⑥
He et al. (22)	China	Case–control	102	DFU	≥60	MNA-SF	Inpatient Department	100.0	37.25	—	① ④ ⑥ ⑦ ⑧ ⑨ ⑩ ⑪ ⑯
Huang et al. (23)	China	Cross-sectional	208	DFU	≥60	MNA-SF	Inpatient Department	43.2	61.54	—	① ③ ④ ⑥ ⑨ ⑩⑫ ⑭
Dong et al. (24)	China	Cross-sectional	136	DFU	≥65	NRS2002	Inpatient Department	36.0	—	70.58	① ④ ⑧ ⑪ ⑨ ⑩
Ran et al. (25)	China	Cross-sectional	559	DFU	≥60	MNA	Inpatient Department	42.5	69.34	—	① ④ ⑧ ⑪ ⑨ ⑩⑫ ⑮
Shen et al. (26)	China	Cross-sectional	180	T2DM	≥60	MNA	Community	57.2	—	31.7	NA
Jian et al. (27)	China	Cross-sectional	94	DM	≥65	MNA-SF	Inpatient Department	26.5	25.53	30.85	NA
Zhang et al. (28)	China	Cross-sectional	700	T2DM	≥60	MNA	Inpatient Department	52.1	—	78.5	① ④ ⑧ ⑪ ⑬ ⑯
Zhang et al. (29)	China	Cross-sectional	325	T2DM	≥60	MNA-SF	Community	40.6	24.31	—	① ② ③ ④ ⑤ ⑦
Niu et al. (30)	China	Cross-sectional	576	T2DM	≥60	GNRI	Inpatient Department	38.8	—	14.0	① ④
Bian et al. (31)	China	Cross-sectional	361	T2DM	≥60	MNA	Inpatient Department	53.7	23.27	68.70	NA
Lu et al. (32)	China	Cross-sectional	176	DKD	≥60	MNA	Inpatient Department	—	32.95	67.05	NA
Lu et al. (33)	China	Cross-sectional	105	DM	≥60	MNA	Inpatient Department	39.0	12.4	59.0	NA
Chen et al. (34)	China	Case–control	90	DKD	≥60	NRS2002	Inpatient Department	47.7	—	62.22	④ ⑦ ⑬
Zhang et al. (35)	China	Cross-sectional	420	T2DM	≥65	NRS2002	Inpatient Department	55.4	—	35.7	⑫
Ran et al. (36)	China	Cross-sectional	248	T2DM	≥65	MNA-SF	Inpatient Department	38.3	26.21	—	④ ⑨ ⑥
Yang et al. (37)	Korea	Cross-sectional	2,376	DM	≥65	DETERMINE Your Nutritional Health Checklist	Community	60.2	—	41.75	②⑮
Ida et al. (38)	Japan	Cross-sectional	510	DM	≥65	GLIM	Outpatient Department	39.2	21.5	—	NA
Thaenpramun et al. (39)	Thailand	Cross-sectional	287	T2DM	≥60	MNA	Outpatient Department	63.4	—	15.0	NA
Tasci et al. (40)	Turkey	Cross-sectional	215	T2DM	≥65	MNA-SF	Community	70.2	33.0	3.7	③ ⑫
Junaid et al. (41)	Nigeria	Cross-sectional	96	T2DM	≥60	MNA-SF	Inpatient Department	50.0	7.3	42.7	NA
Li et al. (42)	Korea	Cross-sectional	464	DM	69.6 ± 2.96	NSI	Community	100.0	34.69	—	NA
Kimura et al. (43)	Japan	Cohort	1754	T2DM	≥65	GNRI	Inpatient Department	29.8	49.2	—	NA
Kong et al. (44)	China	Cross-sectional	291	T2DM	≥65	MNA	Community	52.9	2.1	33.0	NA
Martin et al. (45)	Spain	Cohort	402	T2DM	≥65	MNA	Community	55.5	77.6	—	NA
Shiroma et al. (46)	Japan	Cross-sectional	479	T2DM	71 [62,77]	GNRI	Outpatient Department	44.8	—	9.0	NA
Saintrain et al. (47)	Brazil	Cross-sectional	246	T2DM	65–94	MNA	Outpatient Department	56.0	3.7	15.9	NA
Kulkarni et al. (48)	India	Cross-sectional	104	DM	≥60	MNA	Community	72.1	16.3	70.2	NA

*Participants*: Diabetic nephropathy (DN); Type 2 diabetes mellitus (T2DM); Diabetic foot ulcer (DFU); Diabetes mellitus (DM); Chronic diseases of diabetes, including diabetic macroangiopathy, diabetic peripheral neuropathy, diabetic retinopathy, diabetic nephropathy and diabetic foot.

*Measurement tools of nutrition status*: Mini nutritional assessment (MNA); Mini-nutritional Assessment short-form (MNA-SF); Nutritional Risk screening 2002 (NRS-2002); Geriatric nutritional risk index (GNRI); Global Leadership Initiative on Malnutrition (GLIM); Nutrition Screening Initiative (NSI); DETERMINE Your Nutritional Health Checklist (DYNHC).

Risk factors: ① age; ②Complicated chronic diseases; ③ polypharmacy; ④ glycosylated hemoglobin(HbA1c); ⑤ regular exercise; ⑥ albumin (ALB); ⑦ Body mass index (BMI); ⑧ duration of diabetes; ⑨ With diabetic foot infection; ⑩Wagner grades 3–5; ⑪ C-reactive protein (CRP) > 10 mg/L; ⑫activities of daily living(ADL); ⑬ Abnormal blood urea nitrogen level (BUN); ⑭ Sleep disorder; ⑮ Depression; ⑯ Unreasonable dietary structure.

NA, not applicable.

a: Prevalence of malnutrition in patients with diabetes; b: Prevalence of malnutrition and risk of malnutrition in patients with diabetes.

### 3.3 Quality assessment

The scores for cross-sectional studies based on the AHRQ criteria were as follows: 6 points (*n* = 15), 7 points (*n* = 8), and 8 points (*n* = 3). Meanwhile, the scores for cohort and case–control studies based on the NOS were as follows: 6 points (*n* = 2) and 7 points (*n* = 2). Detailed quality scores are presented in Tables 2, 3.

**Table 2:** Quality assessment of included studies (cross-sectional studies).

Number	Study	Study design	AHRQ											score
			Whether the source of the data is clear (survey, literature review)	Exposed and non-exposed inclusion and exclusion criteria are listed or refer to previous publications	Whether a time period was given to identify patients	If not population source, study subjects were continuous	Whether the subjective factors of the evaluator cover up other aspects of the research object	Any evaluation performed for quality assurance is described	The rationale for excluding any patient from the analysis was explained	Describe how to evaluate and/or control for confounders	If possible, explain how missing data were handled in the analysis	Patient response rates and completeness of data collection were summarized	If there is follow-up, identify the percentage of incomplete data or follow-up outcomes for the expected patients	
1	Gao et al. (19)	Cross-sectional	1	1	1	1	1	0	Not clear	0	Not clear	1	0	6
2	Ling et al. (20)	Cross-sectional	1	1	0	1	1	1	Not clear	1	Not clear	1	0	7
3	Chu et al. (21)	Cross-sectional	1	1	0	1	1	0	Not clear	1	Not clear	1	0	6
4	Huang et al. (23)	Cross-sectional	1	1	1	1	1	0	Not clear	0	Not clear	1	0	6
5	Dong et al. (24)	Cross-sectional	1	1	0	1	1	1	0	0	1	1	0	7
6	Ran et al. (25)	Cross-sectional	1	1	0	1	1	1	0	0	1	1	0	7
7	Shen et al. (26)	Cross-sectional	1	1	0	1	1	1	0	0	1	0	0	6
8	Jian et al. (27)	Cross-sectional	1	1	1	1	1	1	0	0	0	0	0	6
9	Zhang et al. (28)	Cross-sectional	1	1	1	1	1	1	0	0	1	1	0	8
10	Zhang et al. (29)	Cross-sectional	1	1	1	1	0	1	0	0	1	1	0	7
11	Niu et al. (30)	Cross-sectional	1	1	1	1	1	1	0	0	1	1	0	8
12	Bian et al. (31)	Cross-sectional	1	1	1	1	1	1	0	0	1	1	0	8
13	Lu et al. (32)	Cross-sectional	1	1	1	1	1	1	0	0	0	0	0	6
14	Lu et al. (33)	Cross-sectional	1	1	1	1	1	1	0	0	0	0	0	6
15	Zhang et al. (35)	Cross-sectional	1	1	1	1	1	1	0	1	0	1	0	7
16	Ran et al. (36)	Cross-sectional	1	1	1	1	1	0	0	0	1	0	0	6
17	Yang et al. (37)	Cross-sectional	1	1	1	1	1	0	0	1	0	0	0	6
18	Ida et al. (38)	Cross-sectional	1	1	0	1	1	1	0	1	0	0	0	6
19	Thaenpramun et al. (39)	Cross-sectional	1	1	1	1	1	1	0	1	0	0	0	7
20	Tasci et al. (40)	Cross-sectional	1	1	1	1	1	1	0	1	0	0	0	6
21	Junaid et al. (41)	Cross-sectional	1	1	1	1	1	1	0	0	0	0	0	6
22	LIM et al. (42)	Cross-sectional	1	1	0	1	1	1	0	0	1	0	0	6
23	Kong et al. (44)	Cross-sectional	1	1	0	1	1	1	0	0	1	1	0	7
24	Shiroma et al. (46)	Cross-sectional	1	1	1	1	1	1	0	0	0	1	0	7
25	Saintrain et al. (47)	Cross-sectionalc	1	1	1	1	1	1	0	0	0	0	0	6
26	Kulkarni et al. (48)	Cross-sectional	1	1	1	1	1	1	0	0	0	0	0	6

Quality assessment criteria recommended by the U.S. Agency for Health Care Research and Quality (AHRQ): It includes 11 items, which are answered with “yes,” “no” and “not clear” respectively. For each item, “yes” will be counted as 1 point, otherwise, it will be counted as 0 point. Item 5 is the reverse score item, and the full score is 11 points, ≥ 8 is of high quality, 6–7 is of medium quality, and ≤5 is of low quality.

**Table 3:** Quality assessment of included studies (case–control or cohort studies).

Number	Study	Study design	NOS								Score
			Selection (4 point)				Comparability (2 point)	Exposure/outcome (3 point)			
			What is the representativeness of the exposed group (1 point)	Selection method of non-exposed group (1 point)	Methods for determining exposure factors (1 point)	The outcome that was not observed at the beginning of the study was determined (1 point)	Consideration of comparability between exposed and unexposed groups in design and statistical analysis (2 points)	Whether the study assessed the adequacy of the outcome (1 point)	Was follow-up long enough after the outcome (1 point)	Was follow-up adequate between exposed and unexposed groups (1 point)	
1	He et al. 2020 (22)	Case–control	1	1	1	1	1	1	0	0	6
2	Chen et al.2023 (34)	Case–control	1	1	1	1	1	1	0	0	6
3	Kimura et al.2021 (43)	Cohort	1	1	1	1	1	1	1	0	**7**
4	Martin et al. (45)	Cohort	1	1	1	1	1	1	1	0	**7**

The newcastle-ottawa scale (NOS) was used as a quality assessment tool for cohort studies and case–control studies. The evaluation content of NOS included the selection of research subjects (4 items, 4 points), comparability between groups (2 items, 4 points), and the evaluation of NOS. 2 points), outcome or measurement of exposure factors (3 items, 3 points) 3 aspects, a total of 9 items out of 9 points. A score of ≤3 was defined as low quality literature, 4–6 as medium quality literature, and ≥ 7 as high quality literature.

### 3.4 Quality assessment

**Figure 2:**
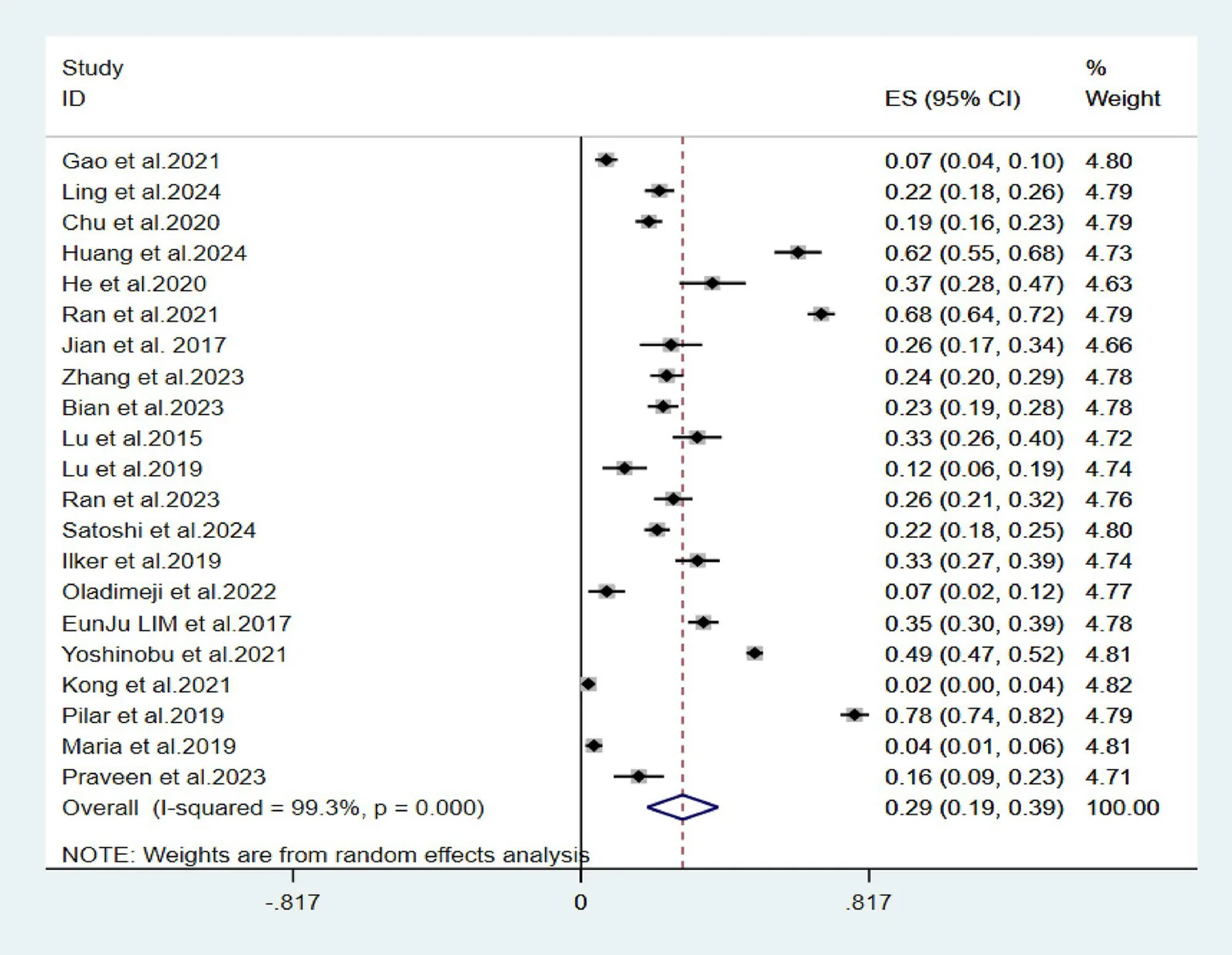
Forest plot of malnutrition prevalence in elderly patients with diabetes. CI, confidence interval; ES, effect size.

Based on 21 studies (19–22, 24, 26, 28, 30–32, 34, 36, 38–41, 43–47), the pooled prevalence rate of malnutrition in elderly patients with diabetes was 29% (95% CI: 0.19–0.39). These 21 studies exhibited significant heterogeneity (*I*^2^ = 99.3%; *p* < 0.001). Subgroup analyses according to nutritional assessment tool, study setting, and presence of chronic complications revealed that the type of assessment tool (such as MNA vs. NRS-2002) and geographic region partially explained the variance (*p* < 0.05), suggesting that methodological and population differences contributed to the high heterogeneity. A random-effects model was used (Figure 2).

### 3.5 Subgroup analysis of malnutrition prevalence

Subgroup analysis according to chronic complications revealed that the prevalence rate of malnutrition in elderly patients with diabetes with chronic complications (50.6%) was significantly higher than that in elderly patients with diabetes without chronic complications (20.4%) (*p* < 0.05) (Table 4).

**Table 4:** Subgroups analysis of malnutrition.

Subgroups		(n)	Malnutrition (%)	95% CI	*I^2^* (%)	*p* value	*p* value
Chronic complications	Yes	4 (22, 23, 25, 32)	50.6	0.323–0.687	99.10	<0.001	0.004
	No	17 (19–21, 27, 29, 31, 33, 36, 38, 40–45, 47, 48)	20.4	0.116–0.309	99.036	<0.001	
Measurement tool	MNA	8 (25, 31–33, 44, 45, 47, 48)	26.20	0.070–0.521	99.377	<0.001	0.930
	MNA-SF	10 (19–23, 27, 29, 36, 40, 41)	25.10	0.166–0.346	96.059	<0.001	
Site of study	Inpatient department	12 (19, 21–23, 25, 27, 31–33, 36, 41, 43)	29.5	0.182–0.421	98.524	<0.001	0.225
	Outpatient department	3 (20, 38, 47)	14.5	0.048–0.283	99.20	<0.001	
	Community	6 (29, 40, 42, 44, 45, 48)	28.9	0.089–0.545	99.20	<0.001	

### 3.6 Pooled prevalence of high malnutrition risk

**Figure 3:**
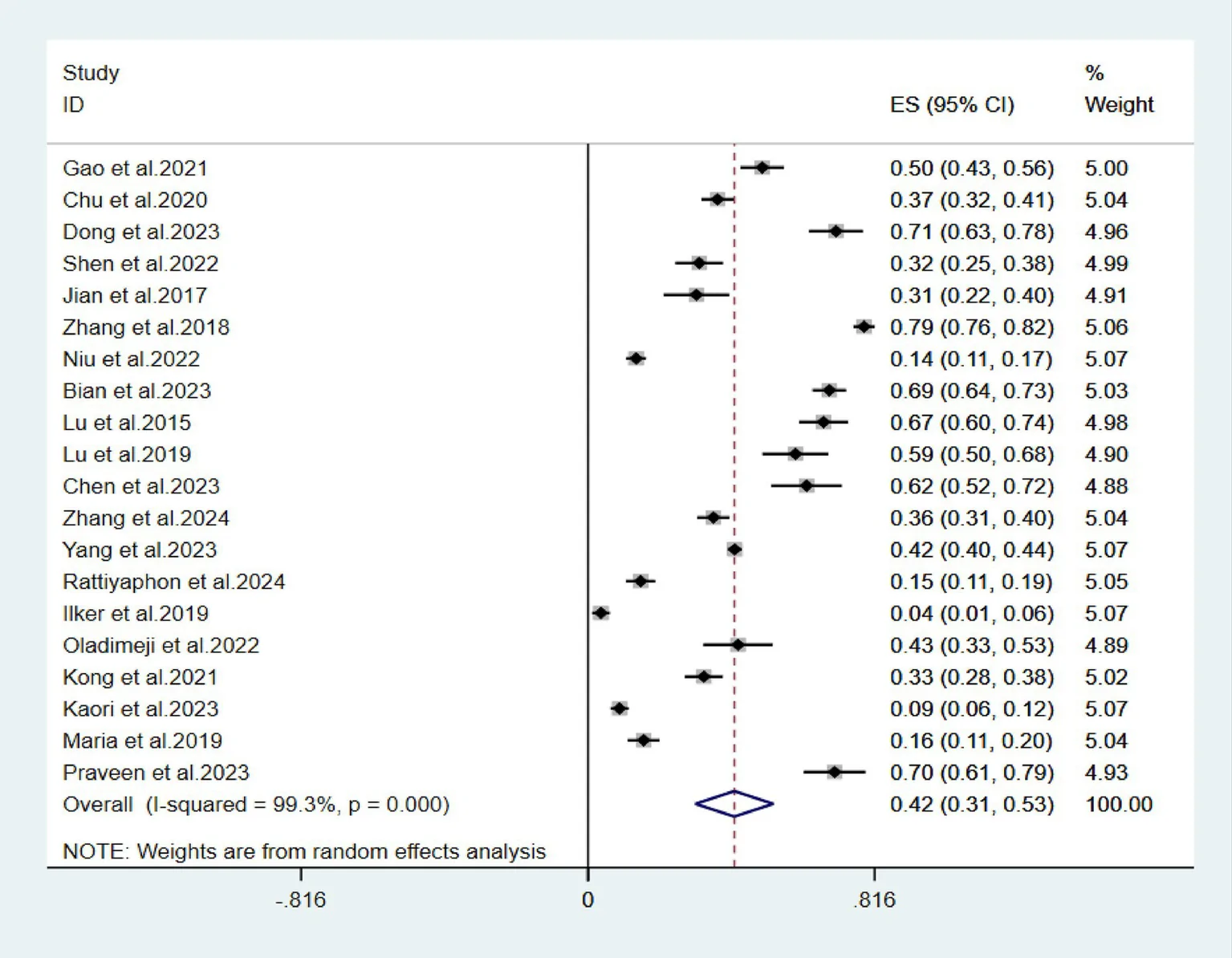
Forest plot of high malnutrition risk prevalence in elderly patients with diabetes. CI, confidence interval; ES, effect size. This analysis included 20 studies reporting at-risk malnutrition.

Based on 20 studies (19, 21, 23, 25–27, 29–33, 35–37, 39, 41–44, 48), the pooled prevalence rate of high malnutrition risk was 42% (95% CI: 0.31–0.53). A random-effects model was used (Figure 3).

### 3.7 Subgroup analysis of high malnutrition risk

Subgroup analysis according to chronic complications demonstrated that the incidence rate of high malnutrition risk in patients with chronic complications (67.2%) was higher than that in patients without chronic complications (35.8%) (*p* < 0.05) (Table 5).

**Table 5:** Subgroups analysis of at-risk for malnutrition.

Subgroups		(n)	Prevalence (%)	95% CI	*I^2^* (%)	*p* value	*p* value
Chronic complications	Yes	3 (24, 32, 34)	67.2	62.5–71.7	0	1	<0.001
	No	17 (19, 21, 26–28, 30, 31, 33, 35, 37, 44, 46–48, 39–41)	35.8	24.9–47.4	98.94	<0.001	
Measurement tool	NRS-2002	3 (24, 34, 35)	56.1	0.319–0.789	0	1	<0.001
	MNA	9 (26, 28, 31–33, 39, 44, 47, 48)	48.3	0.296–0.672	98.88	<0.001	
	MNA-SF	5 (19, 21, 27, 40, 41)	30.6	0.134–0.512	97.789	<0.001	
	GNRI	2 (30, 46)	11.6	0.098–0.136	0	<0.001	
Site of study	Inpatient department	10 (19, 21, 24, 27, 28, 30, 35, 41, 31–)	51.1	0.364–0.659	98.667	<0.001	<0.001
	Community	5 (26, 37, 40, 44, 48)	33.6	0.171–0.525	98.40	<0.001	
	Outpatient department	3 (39, 46, 47)	12.9	0.085–0.180	0	<0.001	

### 3.8 Risk factors associated with malnutrition

This study examined 16 potential risk factors of malnutrition. BMI and blood urea nitrogen (BUN) were not significantly associated with malnutrition (*p* > 0.05). Thus, the following 14 factors were identified as significant risk factors: decreased serum albumin (ALB), dependence or impairment in activities of daily living (ADL), lack of regular exercise, elevated glycated hemoglobin (HbA1c) levels, C-reactive protein (CRP) levels of >10 mg/L, advanced age, prolonged diabetes duration, Wagner grade 3–5 foot ulcers, foot ulcer infections, chronic diseases, polypharmacy, sleep disorders, depression, and unreasonable dietary structure (Table 6).

**Table 6:** Risk factors of malnutrition in elderly patients with diabetes.

Risk factors	Number of included studies(*n*)	Heterogeneity		Model	Results of Meta-analysis		
		*I^2^* (%)	*p* value		Odds ratio	95% CI	*p* value
Sleep disorder	2 (20, 23)	0	0.693	Fixed	1.548	1.148–2.087	0.004
Age, years	9 (20–25, 28–30)	85.6	<0.001	Random	2.293	1.511–3.480	<0.001
Complicated chronic diseases	4 (20, 21, 29, 37)	72.4	0.012	Random	2.421	1.563–3.750	<0.001
polypharmacy	4 (20, 23, 29, 40)	0	0.913	Fixed	3.088	2.172–4.392	<0.001
HbA1c, %	11 (20–25, 28–30, 34, 36)	87	<0.001	Random	2.232	1.659–3.002	<0.001
regular exercise	3 (20, 21, 29)	40.7	0.185	Fixed	0.247	0.122–0.498	<0.001
ALB, g/L	6 (20–23, 34, 36)	95.4	<0.001	Random	0.448	0.273–0.736	0.002
BMI, kg/m2	4 (22, 29, 34, 36)	91.9	<0.001	Random	0.738	0.398–6.368	0.334
Duration of diabetes, years	4 (22, 24, 25, 28)	11.9	0.333	Fixed	2.630	1.907–3.627	<0.001
CRP, > 10 mg/L	4 (22, 24, 25, 28)	61.8	0.049	Random	1.923	1.038–2.826	0.001
Wagner grades 3–5	4 (22–25)	22.4	0.276	Fixed	4.881	3.008–7.920	<0.001
With diabetic foot infection	5 (22–25, 36)	66.3	0.018	Random	3.057	1.790–5.219	<0.001
activities of daily living(ADL)	4 (23, 25, 29, 40)	88.7	<0.001	Random	0.647	0.465–0.899	0.009
Unreasonable dietary structure	2 (22, 28)	0	0.486	Fixed	3.451	2.329–5.113	<0.001
Depression	2 (25, 37)	0	0.451	Fixed	3.686	2.699–5.035	<0.001
Abnormal blood urea nitrogen level	2 (28, 34)	93.6	<0.001	Random	1.523	0.678–3.423	0.309

Protective effect of exercise refers to regular aerobic and/or resistance training.

### 3.9 Sensitivity analysis and publication bias

**Figure 4:**
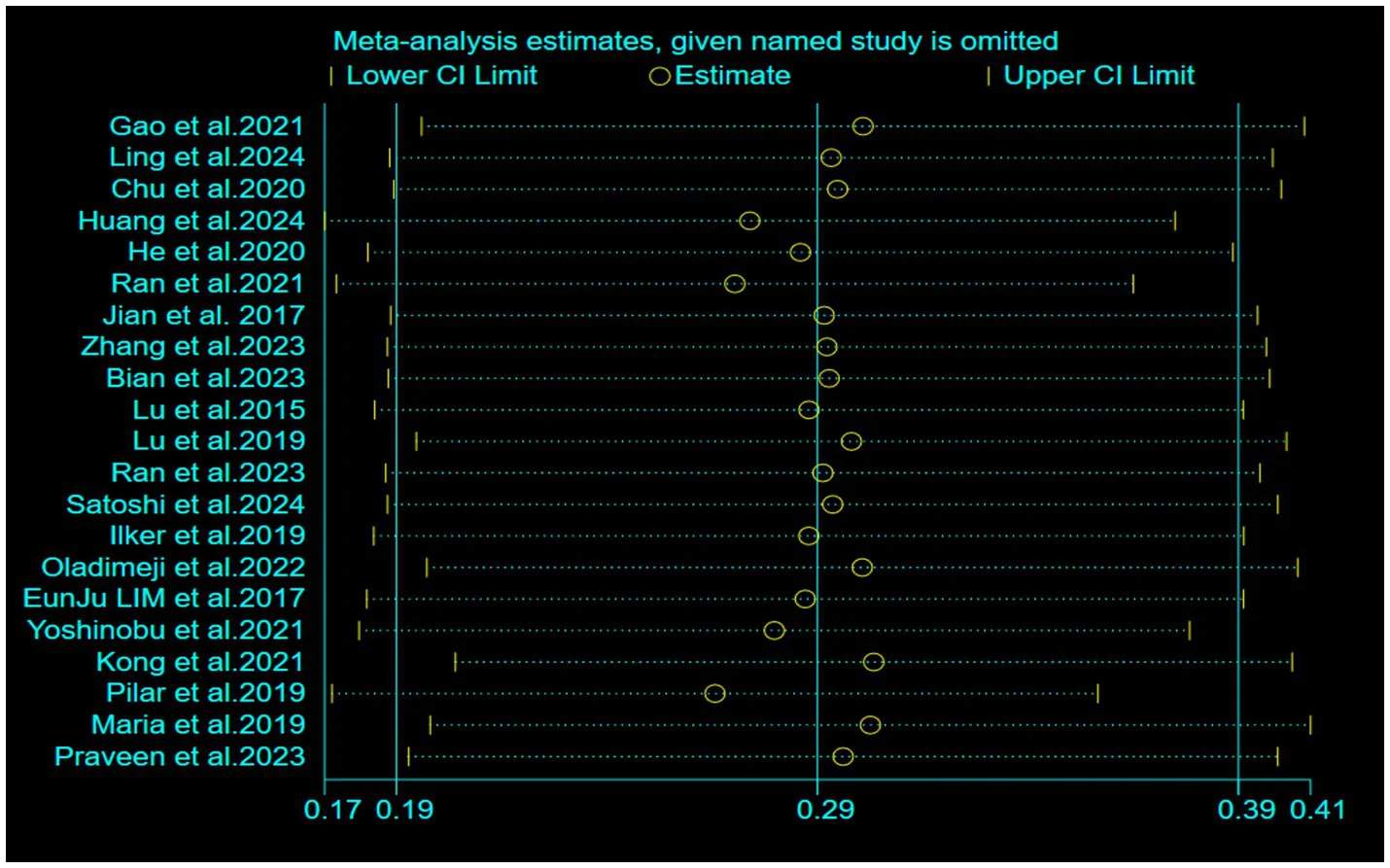
Sensitivity analysis of malnutrition prevalence.

**Figure 5:**
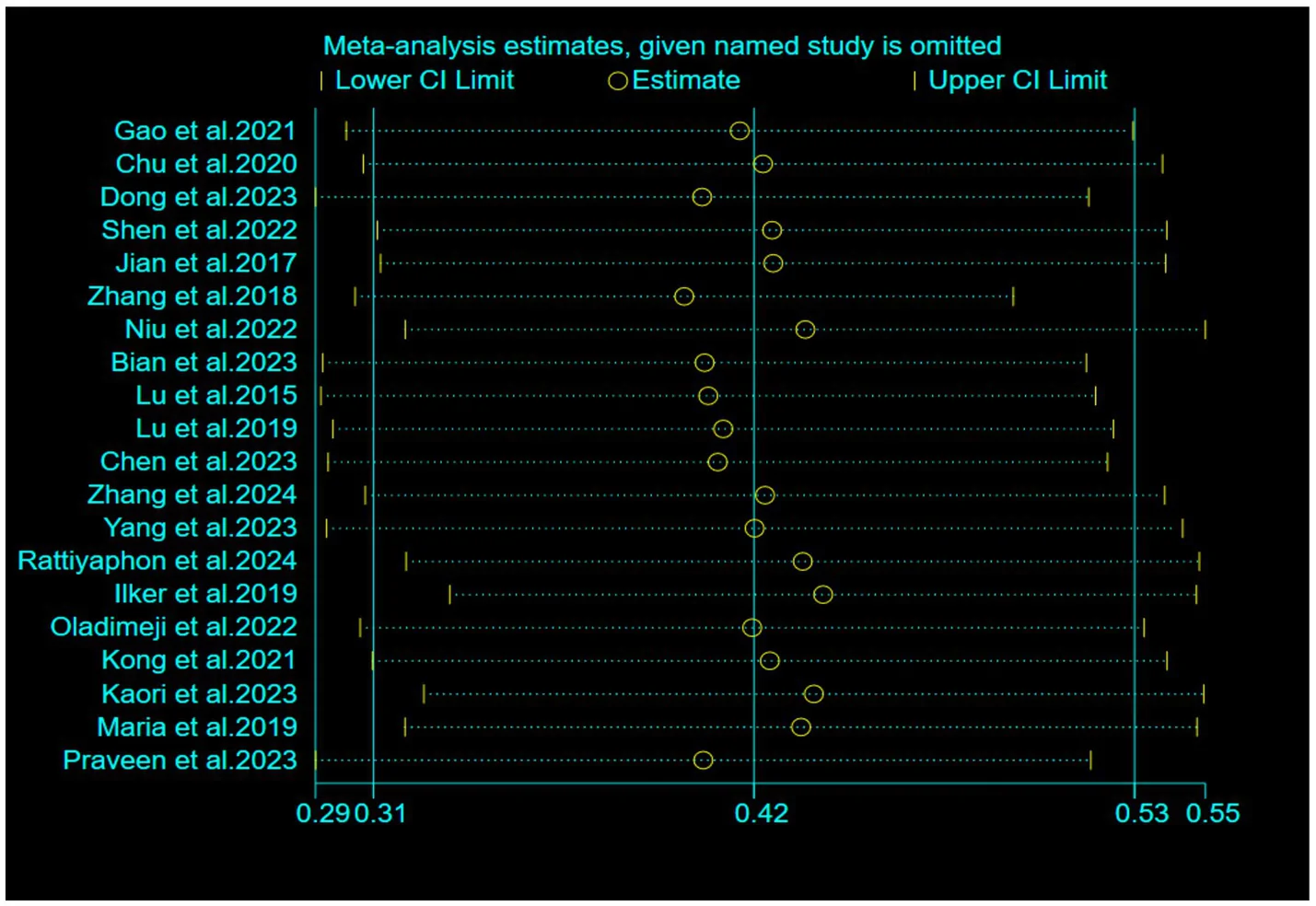
Sensitivity analysis of high malnutrition risk prevalence.

**Figure 6:**
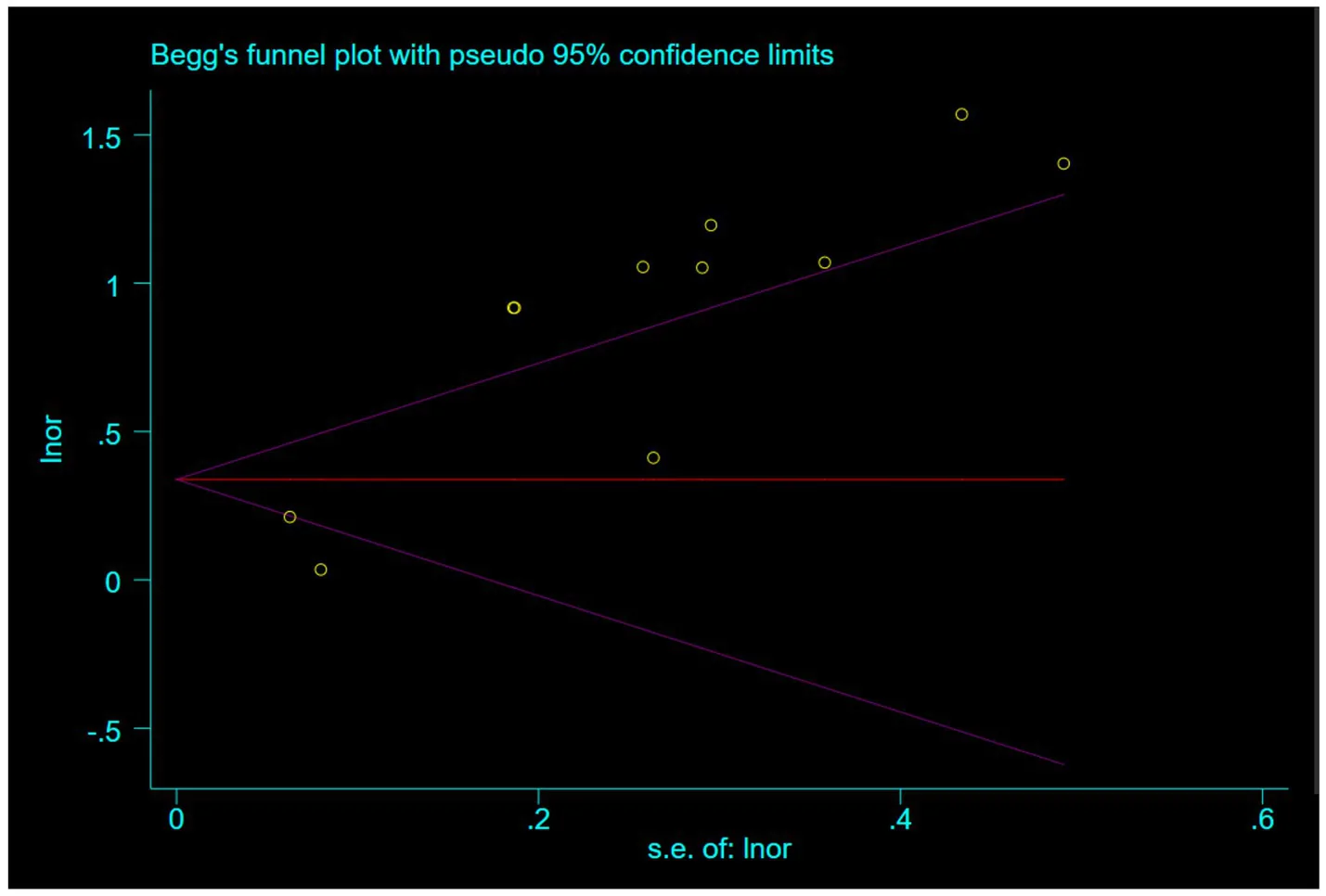
Funnel plot for assessing publication biases.

**Figure 7:**
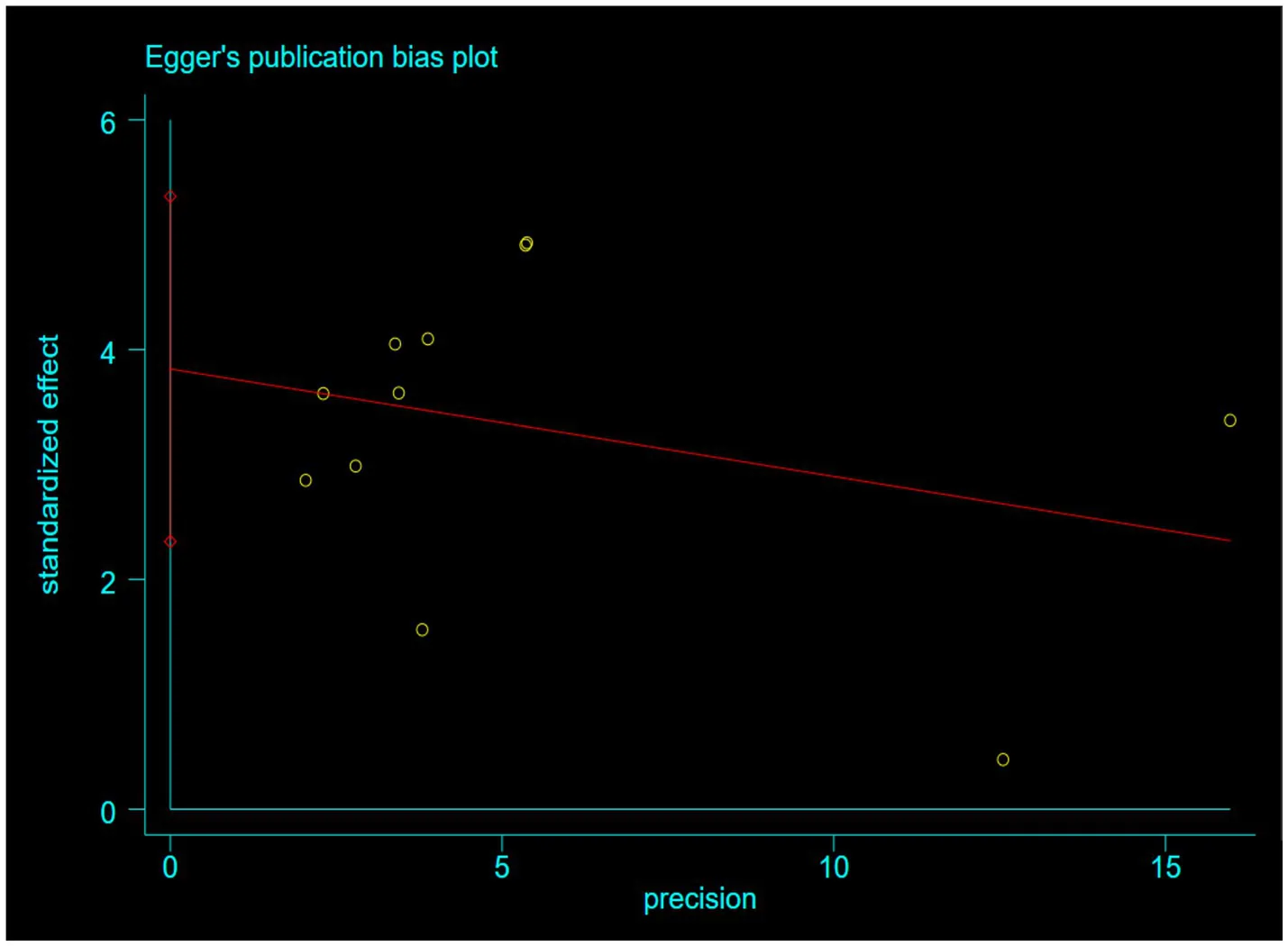
Egger’s publication bias plot.

**Figure 8:**
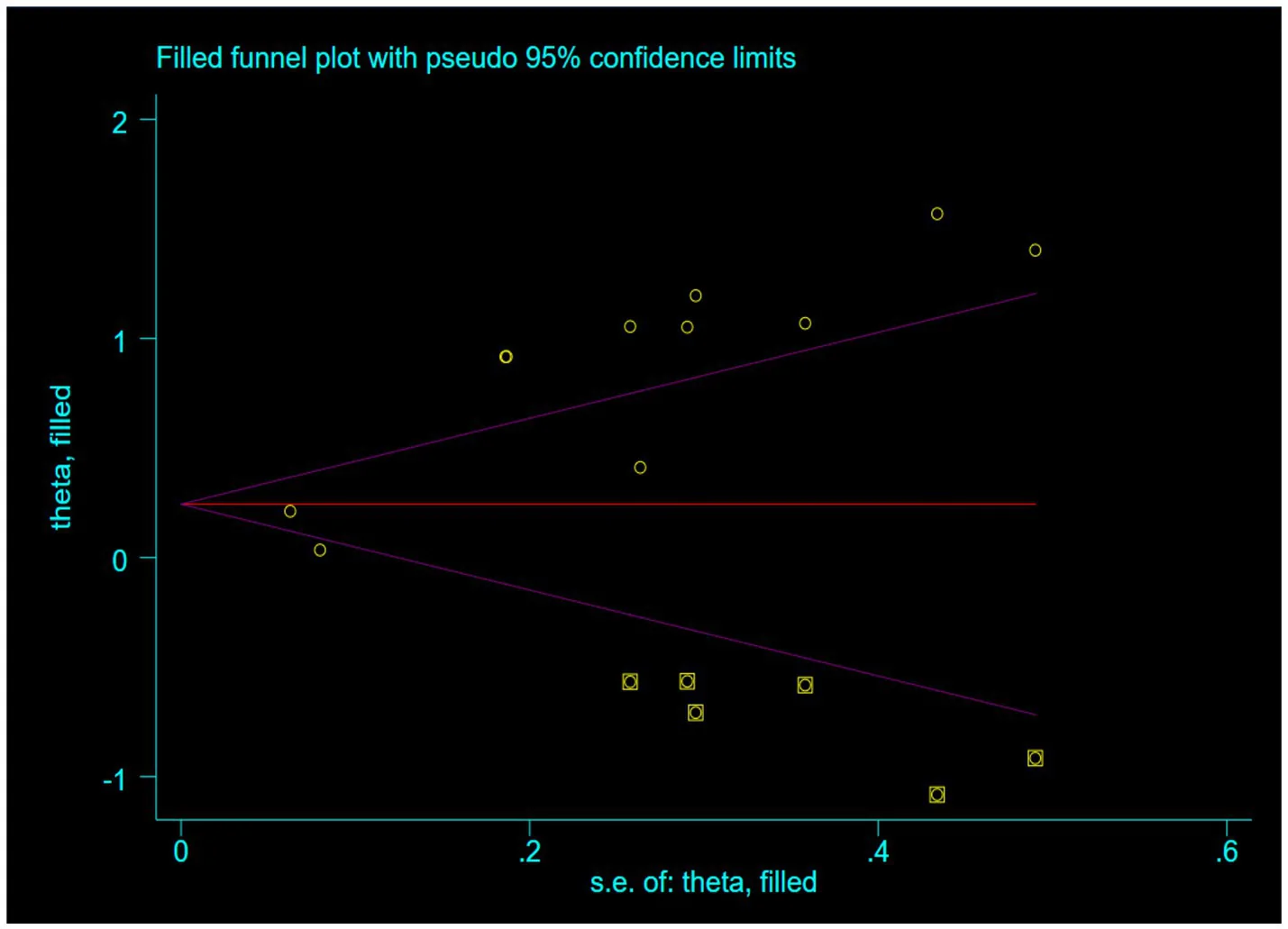
Trim-and-fill analysis for publication bias adjustment.

Sensitivity analysis revealed no significant change in the pooled effects after sequentially excluding individual studies, indicating stable results (Figures 4, 5). Egger’s test revealed publication bias for HbA1c (reported in >10 studies) (*p* < 0.05; Figures 6, 7). The trim-and-fill method confirmed that the significance did not change after adjustment, suggesting that publication bias did not affect the stability of the results (Figure 8).

## 4 Discussion

This study included 30 studies involving 12,564 cases from nine countries. More than two-thirds of the included studies were from Asian countries (China, South Korea, Japan, Thailand, and India). The highest proportion of studies was from China. This indicates that the findings of this study mainly reflect the characteristics of Asian elderly patients with diabetes. Thus, caution must be exercised when extrapolating the findings to other ethnic groups and regions (such as Europe, North America, and Africa). The overall prevalence rate of malnutrition was 29%, while the prevalence rate of high malnutrition risk was 42%. The risk factors for malnutrition identified in this study were as follows: decreased serum ALB, impaired ADL, lack of regular exercise, elevated HbA1c, CRP levels of >10 mg/L, advanced age, prolonged diabetes duration, Wagner grade 3–5 foot ulcers, foot ulcer infection, chronic diseases, polypharmacy, sleep disorders, depression, and unreasonable dietary structure.

Aberrant BMI and BUN values were not correlated with malnutrition, which was not consistent with the findings of previous studies. BMI is not a reliable nutritional indicator in elderly patients with diabetes, which can be attributed to several reasons. Sarcopenia is common in elderly patients with diabetes. Fat mass cannot be differentiated from lean mass based on BMI. Thus, patients with healthy or even high BMI may exhibit severe muscle depletion and malnutrition. Additionally, edema and the coexistence of obesity with malnutrition are frequent in these patients, which can lead to the overestimation or underestimation of true nutritional risk. Furthermore, age-related height loss and spinal kyphosis affect BMI accuracy (10). Therefore, BMI should be used cautiously as a stand-alone tool in elderly patients with diabetes. Combining BMI with comprehensive tools, such as MNA or GNRI is recommended.

Patients with chronic complications exhibited increased prevalence of malnutrition and high malnutrition risk, which is consistent with the findings of a meta-analysis examining adult patients with diabetes (49). The prevalence rates of malnutrition and high malnutrition risk in elderly patients (50.6 and 67.2%, respectively) were higher than those in adults (49 and 51%, respectively). Late-stage diabetes is often characterized by chronic complications with prolonged hypermetabolism promoting energy and protein consumption, which leads to malnutrition (50). Hospitalized patients exhibited higher malnutrition risk than outpatients and community-dwelling patients. This can be attributed to increased disease severity and poor physical function. Subgroup analyses according to the assessment tool revealed differences in prevalence estimates, highlighting the need for a specific, reliable malnutrition screening tool for patients with diabetes.

Various validated tools have been developed for examining the malnutrition status. The validity and applicability of these tools vary depending on the clinical setting and patient characteristics. In the included studies, MNA and MNA-SF were the most frequently used tools, followed by GNRI and NRS-2002. This study did not directly compare the predictive validity of these tools. These validated tools can be used for routine clinical practice based on local availability and patient population.

ADL measure the ability of an individual to function in daily life, ranging from completely independent to heavily dependent. This measure includes instrumental ADL and basic ADL, such as eating, dressing, bathing, and transferring, which reflect functional status and self-care ability (51–53). Impaired ADL reduce self-care ability and physical function, promoting or aggravating malnutrition. Dependence in ADL may limit access to food, meal preparation, self-feeding, and adherence to dietary recommendations. Conversely, malnutrition leads to the loss of muscle mass and body cell mass, impairing physical function. Impaired ADL and malnutrition reinforce the effects of each other through frailty and sarcopenia, leading to loss of independence. Therefore, ADL should be recognized as both an important risk marker and a potential intervention target. Healthcare providers must routinely assess ADL in elderly patients with diabetes and provide necessary support (such as home care, assistive devices, caregiver education) to maintain functional ability and prevent malnutrition.

Regular aerobic exercises, such as walking, cycling, Tai Chi, and Baduanjin, and resistance training, exert protective effects against malnutrition. Exercise helps control fasting blood glucose, enhance muscle strength, improve physical function, oxygen consumption and muscle endurance, flexibility and balance, gastrointestinal activity, and appetite and delay frailty progression, ensuring nutritional intake (54). Traditional Chinese mind–body exercises, such as Tai Chi and Baduanjin, which combine aerobic and balance training, provide significant benefits for blood glucose control, muscle strength, physical function, appetite, and mental health, alleviating depressive symptoms and improving quality of life in patients with type 2 diabetes (55, 56). Combining regular aerobic exercise with resistance training is recommended as an effective strategy to prevent malnutrition.

Serum ALB levels have been widely studied for malnutrition diagnosis. Hypoalbuminemia (serum ALB < 3.5 g/dL) is traditionally considered an indicator of malnutrition. However, two meta-analyses revealed that the serum ALB threshold of 3.5 g/dL along with multiple nutritional screening tools (MNA, NRS-2002, MNA-SF, and GNRI) may lead to the underdiagnosis of malnutrition (57, 58). This threshold value must be further validated for elderly patients.

In this study, increased HbA1c levels, which disrupt glucose and amino acid metabolism (59), were associated with malnutrition. Advanced age was also a risk factor, which may be due to an age-related reduction in intestinal plexus nerve cell functions, adversely affecting digestion and absorption (60, 61). Wagner grade 3–5 foot ulcers, foot ulcer infection, and CRP levels >10 mg/L were also risk factors for malnutrition. Severe infection induces an inflammatory response that accelerates protein breakdown (62). Prolonged diabetes duration increases malnutrition risk due to progressive islet dysfunction and protein catabolism (63, 64).

Sleep disorders and depression were bidirectionally associated with malnutrition. However, cross-sectional studies cannot establish causality. Poor sleep and depression may reduce appetite and promote irregular eating, while malnutrition may affect neurotransmitter synthesis and energy metabolism, exacerbating depression and sleep disorders (65, 66). Unreasonable dietary structure (such as high carbohydrate and low protein and fiber) is an important modifiable risk factor for malnutrition. Prospective cohort studies are warranted to clarify the causal relationships.

Polypharmacy (concomitant use of ≥ 5 medications), which is common in elderly patients with diabetes, is associated with adverse effects that can exacerbate malnutrition. Anticholinergic drugs can promote dry mouth, dysgeusia, constipation, and cognitive impairment, reducing food intake and ADL (67, 68). Psychotropic medications may induce sedation and cognitive decline (69). Metformin is associated with gastrointestinal disturbances and may interfere with vitamin B₁₂ absorption. Sulfonylureas and insulin can lead to hypoglycemia in frail elderly patients (69). Cardiovascular drugs (digoxin and amiodarone) may cause anorexia, nausea, and taste alteration (70). Non-steroidal anti-inflammatory drugs and antibiotics can damage the gastrointestinal mucosa, leading to malabsorption (71). Clinicians should regularly review medication regimens, deprescribe when possible, and consider alternative therapies to mitigate nutritional risks associated with polypharmacy.

## 5 Limitations and implications

### 5.1 Limitations

This meta-analysis has several limitations. The pooled prevalence estimates were associated with high heterogeneity, which may be due to the differential nutritional assessment tools, study populations, and geographic regions in different studies. Subgroup analyses did not fully explain the heterogeneity. Most included studies were cross-sectional, limiting causal inferences. The number of studies evaluating some factors (such as BUN and BMI) was small, affecting robustness. Publication bias was detected for HbA1c-related outcomes, suggesting potential overestimation. The predominance of Asian populations (over two-thirds of studies) limits generalizability to other ethnic groups and regions. Risk factor analyses may be affected by differences in the adjustment for confounders across studies. Many included studies were cross-sectional with varying covariate sets, which may influence pooled estimates for factors, such as HbA1c, depression, and polypharmacy. The exclusion of non-English and non-Chinese databases may introduce language bias.

### 5.2 Implications for practice and research

The findings of this study highlight the high burden of malnutrition and high malnutrition risk in elderly patients with diabetes, especially those with chronic complications, polypharmacy, or functional decline. Routine nutritional screening using validated tools should be integrated into geriatric diabetes care. Multidisciplinary interventions, including individualized dietary plans, regular physical activity (such as Tai Chi and resistance training), and medication reviews, are essential to mitigate malnutrition risk. Future studies should focus on developing diabetes-specific nutritional screening tools and conducting longitudinal studies to establish causal relationships and evaluate intervention effectiveness.

## 6 Conclusion

This study summarized the prevalence of malnutrition and high malnutrition risk in elderly patients with diabetes and identified 14 related risk factors. The studies included in the analysis were of high quality with reliable results. Healthcare providers can use these findings to develop early intervention measures for elderly patients with diabetes with malnutrition or high malnutrition risk. However, the number of studies included in the risk factor analysis was small. Additionally, the diversity of nutritional measurement tools increased heterogeneity, affecting the results. Thus, the findings should be interpreted with caution. Further studies are needed to verify and expand these results.
