# Supplementary material for: Prevalence and risk factors of malnutrition in elderly patients with diabetes mellitus: a meta-analysis and systematic review
Source: Front Nutr. 2026 Aug 10;13:1783774. doi: 10.3389/fnut.2026.1783774 (PMC13501966; doi:10.3389/fnut.2026.1783774)
Supplement: Supplementary file 1 [file Table_1.docx]

**Supplementary Materials 1	Search strategy**

|  | **Wanfang** | **Results** |
| --- | --- | --- |
| #1 | 主题:(糖尿病 OR 2型糖尿病 OR 1型糖尿病) |  |
| #2 | 主题:(营养不良 OR 营养失调 OR 营养障碍 OR 营养缺乏 OR 营养风险 OR 营养状况 OR 营养状态) |  |
| #3 | 主题:(危险因素 OR 影响因素 OR 相关因素 OR 患病率 OR 发病率 OR 发生率 OR 流行病学) |  |
| #4 | **#1 AND #2 AND #3** | **2211** |

|  | **CNKI** | **Results** |
| --- | --- | --- |
| #1 | （篇关摘：糖尿病 + 1型糖尿病 + 2型糖尿病） |  |
| #2 | （篇关摘：营养不良 + 营养失调 + 营养障碍 + 营养缺乏 + 营养不良风险 + 营养风险 + 营养状况 + 营养状态） |  |
| #3 | （篇关摘：危险因素 + 影响因素 + 相关因素 + 患病率 + 发病率 + 发生率 + 流行病学） |  |
| #4 | **#1 AND #2 AND #3** | **2569** |

|  | **VIP** | **Results** |
| --- | --- | --- |
| #1 | (题名或关键词=糖尿病 OR 题名或关键词=1型糖尿病 OR 题名或关键词=2型糖尿病) |  |
| #2 | (题名或关键词=营养不良 OR 题名或关键词=营养失调OR 题名或关键词=营养下降OR 题名或关键词=营养障碍OR 题名或关键词=营养缺乏OR 题名或关键词=营养不良风险OR 题名或关键词=营养风险OR 题名或关键词=营养状况OR 题名或关键词=营养状态) |  |
| #3 | (题名或关键词=危险因素 OR 题名或关键词=影响因素OR 题名或关键词=相关因素OR 题名或关键词=高危因素OR 题名或关键词=患病率OR 题名或关键词=发病率OR 题名或关键词=发生率OR 题名或关键词=流行病学) |  |
| #4 | **#1 AND #2 AND #3** | **94** |

|  | **Sinomed** | **Results** |
| --- | --- | --- |
| #1 | 糖尿病[核心字段] OR 2型糖尿病[核心字段] OR 1型糖尿病[核心字段] |  |
| #2 | 营养不良[核心字段] OR 营养失调[核心字段] OR 营养下降[核心字段] OR 营养障碍[核心字段] OR 营养缺乏[核心字段] OR 营养不良风险[核心字段] OR 营养风险[核心字段] OR 营养状况[核心字段] OR 营养状态[核心字段] |  |
| #3 | 危险因素[核心字段] OR 影响因素[核心字段] OR 相关因素[核心字段] OR 高危因素[核心字段] OR 患病率[核心字段] OR 发病率[核心字段] OR 发生率[核心字段] OR 流行病学[核心字段] |  |
| #4 | **#1 AND #2 AND #3** | **768** |

|  | **Pubmed** | Results |
| --- | --- | --- |
| #1 | "Diabetes Mellitus"[Mesh] OR "Diabetes Mellitus"[Title/Abstract] OR Diabetes[Title/Abstract] OR Diabetic[Title/Abstract] OR "type 2 diabetes"[Title/Abstract] OR T2DM[Title/Abstract] OR Diabet*[Title/Abstract] OR "type 1 diabetes"[Title/Abstract] OR T1DM[Title/Abstract] |  |
| #2 | Malnutrition[Mesh] OR "malnutrition"[Title/Abstract] OR "nutritional deficiency"[Title/Abstract] OR "nutritional assessment"[Title/Abstract] OR "nutritional disorders"[Title/Abstract] OR "nutritional status"[Title/Abstract] OR "malnutrition screening"[Title/Abstract] OR "innutrition"[Title/Abstract] OR "nutritional risk"[Title/Abstract] |  |
| #3 | "Risk factors"[Mesh] OR "Risk factors"[Title/Abstract] OR "influence factors"[Title/Abstract] OR "associated factors"[Title/Abstract] OR "dangerous factors"[Title/Abstract] OR Incidence[Mesh] OR Incidence[Title/Abstract] OR Prevalence[Title/Abstract] OR Epidemiology[Mesh] OR Epidemiology[Title/Abstract] OR Frequency[Title/Abstract] |  |
| #4 | **#1 AND #2 AND #3** | 2417 |

|  | **Web of science** | Results |
| --- | --- | --- |
| #1 | TS=("Diabetes Mellitus" OR Diabetes OR Diabetic OR "type 2 diabetes" OR T2DM OR Diabet* OR "type 1 diabetes" OR T1DM) |  |
| #2 | TS=(Malnutrition OR "nutritional deficiency" OR "nutritional assessment" OR "nutritional disorders" OR "nutritional status" OR "malnutrition screening" OR "innutrition" OR "nutritional risk") |  |
| #3 | TS=("Risk factors" OR "influence factors" OR "associated factors" OR "dangerous factors" OR Incidence OR Prevalence OR Epidemiology OR Frequency) |  |
| #4 | **#1 AND #2 AND #3** | 2328 |

|  | **Medline** | Results |
| --- | --- | --- |
| #1 | MeSH[Diabetes Mellitus] OR ("Diabetes Mellitus" OR Diabetes OR Diabetic OR "type 2 diabetes" OR T2DM OR Diabet* OR "type 1 diabetes" OR T1DM):ti,ab,kw |  |
| #2 | MeSH [Malnutrition] OR ("Malnutrition" OR "nutritional deficiency" OR "nutritional assessment" OR "nutritional disorders" OR "nutritional status" OR "malnutrition screening" OR "innutrition" OR "nutritional risk"):ti,ab,kw |  |
| #3 | MeSH [Risk Factors] OR ("Risk factors" OR "influence factors" OR "associated factors" OR "dangerous factors" OR Incidence OR Prevalence OR Epidemiology OR Frequency):ti,ab,kw |  |
| #4 | **#1 AND #2 AND #3** | 482 |

|  | **Embase** | Results |
| --- | --- | --- |
| #1 | ‘Diabetes Mellitus’:ab,ti OR Diabetes:ab,ti OR Diabetic:ab,ti OR ‘type 2 diabetes’:ab,ti OR T2DM:ab,ti OR Diabet*:ab,ti OR ‘type 1 diabetes’:ab,ti OR T1DM:ab,ti |  |
| #2 | ‘Malnutrition’:ab,ti OR ‘nutritional deficiency’:ab,ti OR ‘nutritional assessment’:ab,ti OR ‘nutritional disorders’:ab,ti OR ‘nutritional status’:ab,ti OR ‘malnutrition screening’:ab,ti OR ‘innutrition’:ab,ti OR ‘nutritional risk’:ab,ti |  |
| #3 | ‘Risk factors’:ab,ti OR ‘influence factors’:ab,ti OR ‘associated factors’:ab,ti OR ‘dangerous factors’:ab,ti OR Incidence/exp OR Incidence:ab,ti OR Prevalence:ab,ti OR Epidemiology:ab,ti OR Frequency:ab,ti |  |
| #4 | **#1 AND #2 AND #3** | 1187 |

|  | **EBSCO** | Results |
| --- | --- | --- |
| #1 | SU (diabetes mellitus type 2 or Diabetes Mellitus or Diabetes or T2DM or type 1 diabetes or T1DM) |  |
| #2 | SU (malnutrition or undernutrition or undernourishment or nutritional deficiency or nutritional assessment or nutritional disorders or nutritional status or malnutrition screening or innutrition or nutritional risk) |  |
| #3 | SU (risk factors or influence factors or associated factors or dangerous factors or Incidence/exp or Incidence or Prevalence or Epidemiology or Frequency) |  |
| #4 | **#1 AND #2 AND #3** | 527 |
